# Randomized Controlled Trial of Ultrasonic Propulsion–Facilitated Clearance of Residual Kidney Stone Fragments vs Observation

**DOI:** 10.1097/JU.0000000000004186

**Published:** 2024-08-15

**Authors:** Mathew D. Sorensen, Barbrina Dunmire, Jeff Thiel, Bryan W. Cunitz, Barbara H. Burke, Branda J. Levchak, Christina Popchoi, Arturo E. Holmes, John C. Kucewicz, M. Kennedy Hall, Manjiri Dighe, Jessica C. Dai, Fionnuala C. Cormack, Ziyue Liu, Michael R. Bailey, Michael P. Porter, Jonathan D. Harper

**Affiliations:** 1 Department of Urology, University of Washington School of Medicine, Seattle, Washington; 2 Division of Urology, Veterans Affairs Puget Sound Health Care System, Seattle, Washington; 3 Center for Industrial and Medical Ultrasound, Applied Physics Laboratory, University of Washington, Seattle, Washington; 4 Institute of Translational Health Sciences, University of Washington, Seattle, Washington; 5 Seattle Institute for Biomedical and Clinical Research, Veterans Affairs Puget Sound Health Care System, Seattle, Washington; 6 Department of Emergency Medicine, University of Washington School of Medicine, Seattle, Washington; 7 Department of Radiology, University of Washington School of Medicine, Seattle, Washington; 8 EvergreenHealth Urology Care, Kirkland, Washington; 9 Division of Nephrology, Department of Medicine, University of Washington, Seattle, Washington; 10 Department of Biostatistics and Health Data Science, Indiana University School of Medicine, Indianapolis, Indiana

**Keywords:** kidney stones, ultrasound, lithotripsy

## Abstract

**Purpose:**

Ultrasonic propulsion is an investigational procedure for awake patients. Our purpose was to evaluate whether ultrasonic propulsion to facilitate residual kidney stone fragment clearance reduced relapse.

**Materials and Methods:**

This multicenter, prospective, open-label, randomized, controlled trial used single block randomization (1:1) without masking. Adults with residual fragments (individually ≤5 mm) were enrolled. Primary outcome was relapse as measured by stone growth, a stone-related urgent medical visit, or surgery by 5 years or study end. Secondary outcomes were fragment passage within 3 weeks and adverse events within 90 days. Cumulative incidence of relapse was estimated using the Kaplan-Meier method. Log-rank test was used to compare the treatment (ultrasonic propulsion) and control (observation) groups.

**Results:**

The trial was conducted from May 9, 2015, through April 6, 2024. Median follow-up (interquartile range) was 3.0 (1.8-3.2) years. The treatment group (n = 40) had longer time to relapse than the control group (n = 42; *P* < .003). The restricted mean time-to-relapse was 52% longer in the treatment group than in the control group (1530 ± 92 days vs 1009 ± 118 days), and the risk of relapse was lower (hazard ratio 0.30, 95% CI 0.13-0.68) with 8 of 40 and 21 of 42 participants, respectively, experiencing relapse. Omitting 3 participants not asked about passage, 24 treatment (63%) and 2 control (5%) participants passed fragments within 3 weeks of treatment. adverse events were mild, transient, and self-resolving, and were reported in 25 treated participants (63%) and 17 controls (40%).

**Conclusions:**

Ultrasonic propulsion reduced relapse and added minimal risk.

**Clinical Trial Registration No.:**

NCT02028559.

Kidney stone surgeries commonly leave fragments.^1,2^ Fragments that remain for 3 months are unlikely to spontaneously pass.^3^ Thirty-three percent to 39% of surgeries are repeated within 120 days as stones remain.^4^ Twenty-one percent to 59% of patients with residual stones return for additional care within 5 years.^1^ Residual fragments remain more often in the lower pole,^5,6^ and individual fragments > 5 mm are more likely to require intervention.^7^

**JU Insight**

**Study Need and Importance**
Approximately 50% of patients with small (≤5 mm) residual renal fragments or stones relapse within 5 years.
**What We Found**
We tested if fragment passage facilitated by an ultrasound-based investigational procedure reduced relapse as measured by stone growth; a stone-related, symptomatic, unscheduled medical visit; or surgery. The multisite, randomized, controlled trial was conducted from May 9, 2015, through April 6, 2024, in the clinic setting with awake participants. Median follow-up (interquartile range) was 3.0 (1.8-3.2) years. The treatment group (n = 40) had a 52% longer time to relapse than the control group (n = 42; *P* < .003) with a restricted mean time-to-relapse of 1530 ± 92 days vs 1009 ± 118 days (Figure). The treatment group had a lower risk of relapse (hazard ratio 0.30, 95% CI 0.13-0.68) with 8 of 40 vs 21 of 42 participants experiencing relapse. Omitting 3 participants not asked about stone passage, 24 treatment (63%) and 2 control (5%) participants passed fragments within 3 weeks of treatment. Adverse events were mild, transient, and self-resolving, and were reported in 25 treated participants (63%) and 17 controls (40%).
**Limitations**
The study was relatively small, and few of the participants were non-White, which attenuates generalization to other groups. The urologists in this study were aware of the participants’ group assignments during continued care; however, the shared patient care model in their practices, care provided by urologists outside the study, and the high rates of preventive evaluation (over 55% of participants taking medical prevention) in the participating centers likely counterbalanced that limitation.
**Interpretation for Patient Care**
Our study showed that removing residual fragments by ultrasonic propulsion reduced relapse. The results demonstrate the benefit of an office-based investigational technology to expel fragments without waiting for the possible need for surgery.

**Figure.**
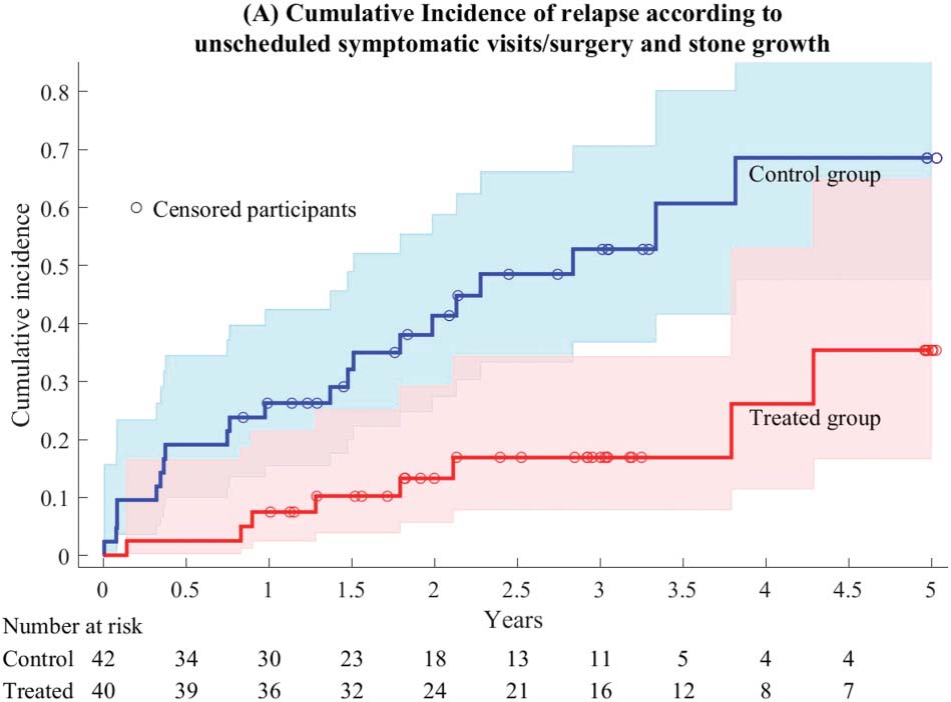
Cumulative incidence of relapse. Shaded areas represent 95% CIs. Small circles represent censored data. Participant relapse data were censored when the participant did not have a relapse before being lost to follow-up or by the end of follow-up.

European urology guidelines recommend postsurgical imaging to detect residual fragments,^1^ and American guidelines recommend offering a second endoscopic surgery to remove fragments.^2^ The discussion in the guidelines focuses on intervening for > 4-mm fragments, and elsewhere the same guidance recommends not intervening and observing small (≤6-mm) asymptomatic stones.^1,2^ Although many fragments will require additional care, prospective controlled studies testing the clinical benefit and risks of intervention are lacking.

Nonsurgical, noninvasive techniques can facilitate reduction in fragment burden.^8-11^ Ultrasonic propulsion is an investigational procedure for awake, unanesthetized patients and uses real-time ultrasound imaging and transcutaneous, focused ultrasound waves to move fragments away from the handheld transducer and toward the ureter to facilitate passage.^12-15^ Prior trials have demonstrated the feasibility, safety, and efficacy of this technology for stone passage.^12-15^

Given the morbidity associated with small residual fragments and lack of concise guidelines regarding the role of surgical intervention, we randomized patients with ≤ 5-mm fragments to no intervention (control group) or to an ultrasonic propulsion procedure to facilitate passage of their fragments (treatment group). Relapse, fragment passage, and adverse events (AEs) were measured to determine whether ultrasonic propulsion would delay relapse compared to a control group.

## MATERIALS AND METHODS

This study was a prospective, investigator-initiated, multicenter, open-label, randomized, controlled trial designed and conducted by the author team under an NIH Certificate of Confidentiality (ClinicalTrials.gov, NCT02028559). The trial was approved by the Institutional Review Boards at the participating institutions (University of Washington IRB No. 00002746, Veterans Affairs IRB No. 01671). The investigational device was made by the University of Washington team, and the investigational device exemption, approved by the US Food and Drug Administration, was sponsored by a university investigator.

### Eligibility Criteria

We included ambulatory patients over age 18 managed by observation with residual fragments (individually ≤5 mm) in the kidney visible on an imaging exam at least 4 weeks after stone surgery. It was confirmed with the referring physician or through the surgical notes that all stones were broken to smaller than 5-mm fragments. Patients on anticoagulants were excluded. No patients with a solitary kidney or a ureteral stone or stent were enrolled.

### Procedures

We performed an ultrasound examination to exclude participants without an unobstructed view of fragments within the target depth (4-10 cm). Participants were then randomized 1:1 to the treatment or control group per one central computer-generated schedule established by the statistician. Randomization block size was 84. The study team was blinded to the treatment assignment status until the participant had passed an ultrasound examination. The treatment group underwent ultrasonic propulsion to reposition and facilitate passage of the residual fragments. Pain was assessed from 0 (no pain) to 10 (maximum pain), and all participants’ skin was observed before screening, after screening, and after the investigational procedure. All participants were provided urine strainers and instructed to watch for fragments.

We contacted participants weekly for 3 weeks to assess for AEs and stone passage. AEs were queried using a script of 10 common surgery- and stone-related symptoms, and any other reported symptoms were documented.^16^ An imaging exam was obtained at least 4 weeks after the procedure to detect obstruction or hematoma. As feasible, the same modality as immediately before the investigational procedure was used to enable comparison. Additional telephone or email follow-up occurred every 6 months for 3 years, and participants’ medical records were reviewed for 5 years to capture stone passage, stone-related urgent medical visits, or stone-related surgeries and the side (right or left) of the event. Annual and additional imaging exams obtained as standard of care were reviewed to identify definitive fragment growth in the study kidney measured by (1) increase in target stone burden (ie, not including when fragments may reposition to appear as fewer larger fragments), (2) increase of target cluster size by ≥ 2 mm, and (3) confirmation of growth in 2 imaging exams or in 1 exam that precipitated surgery or an unscheduled, symptomatic visit. Three independent experts blinded to the participants’ randomization groups adjudicated the imaging (radiologist), safety (nephrologist), and relapse (endourologist) events. All participants were offered standard metabolic evaluation and education on appropriate dietary, medical, and fluid interventions for stone prevention.

### Intervention

The investigational device included a central, in-line commercial imaging transducer with a surrounding hand-held therapy transducer.^12^ The device operated similarly to a diagnostic ultrasound instrument.^17^ Gel and the transducer were placed against the participant’s skin, and the stone was visualized with conventional ultrasound imaging techniques.^15^ When a switch was depressed, 25-millisecond, 350-kHz ultrasound pulses at a maximum pulse intensity of 200 W/cm^2^ were interleaved with the imaging pulses for up to 3 seconds.^12^ The scattering of the ultrasonic propulsion pulses from the fragments caused them to move away from the transducer.^12^ The operator retargeted the fragments and repeated.^13^ The total ultrasound exposure was limited to 5 minutes. Participants were most often positioned in the lateral decubitus position with the bed tilted so their head was down with the transducer on their abdomen under the ribs. However, several patient and transducer positions were used on each participant. A participant in the treatment group could return for a total of 4 treatments. As the participant and study team could see fragments moving or not on the ultrasound screen, there was no masking and no attempt at sham treatment. A stopping rule was in place if any device-related significant AEs were detected.

### Outcomes

The primary outcome was relapse as measured by (1) an urgent, unscheduled, symptomatic medical visit, such as to the emergency department, for stones on the study side, (2) a subsequent surgery for stones on the study side, or (3) stone growth of the targeted residual fragments measured by imaging exams. The prespecified time end point was 5 years following randomization. Follow-up concluded after 5 years or the date of death, withdrawal, or last contact before loss of subject response. There were no cases of death resulting from stone disease and therefore affecting relapse measures. A single participant could only relapse once despite possibly having multiple relapse events. Secondary outcomes were visually observed passage of fragments within 3 weeks and AEs within 90 days.

### Sample Size Calculation

Power calculations and statistical simulations showed that 35 patients per group would provide the trial with a statistical power of 80% with the type 1 error rate of 0.05. We assumed an incidence of relapse of 50% or more in the control group and 15% in the treatment group after 2 years. Weibull regression was used to calculate the sample size to handle a potential mixture of interval and right censoring.^18^ Previous studies that assessed the effects of medical interventions on stone relapse defined as symptomatic events have shown significant effects with sample sizes of 19 to 25 patients per group.^19-21^ In addition, our trial also included radiologic evidence of stone growth, and, thus, we expected that we could detect relapse earlier after surgery than in previous studies. We further increased the overall sample size by 20% and enrolled 14 additional participants to account for study dropout and to increase numbers in demographic subgroups.

### Statistical Analysis

We planned that the overall type 1 error rate would be controlled with the use of single primary hypothesis testing, so no adjustment procedures for multiple comparisons were needed. For the secondary end points, summary statistics and 95% CI were calculated without *P* values.

Demographic and clinical variables were summarized by count and percentage for categorical variables and by median and interquartile range for continuous variables. The cumulative incidence of stone relapse was estimated using the Kaplan-Meier method, and a log-rank test was used to compare the treatment and control groups. We tested the proportional hazards assumption by plotting the log-minus-log survival function against the log(time) and by conducting a resample-based Kolmogorov-type supremum test, neither of which indicated violation of the Cox proportional hazards assumption.^22^ Because of the limited period (5 years or the time to the end of the study), we could not estimate the median time to relapse, so we report the data as the restricted mean time to relapse (the average duration of event-free survival as the area under the survival curve) and standard error; this is smaller than the true (unrestricted) mean time to relapse because of the limited trial period. The difference in the restricted mean time to relapse between the treatment and control groups can be viewed as the mean delay of an event over the trial period. Given that this was a randomized, controlled trial and no imbalance of demographic or clinical variables was observed between the treatment and control groups, no multivariable survival models were fitted. The various event categories that were included in the efficacy results were also summarized by counts and percentages without comparisons between groups because all the patients were not followed for the same duration. A 2-sided *P* value < .05 was considered to indicate statistical significance. Odds ratios for secondary outcomes were calculated by contingency table methods. All analyses were performed with SAS software, version 9.4.

## RESULTS

### Participant Disposition and Baseline Characteristics

**Figure 1.**
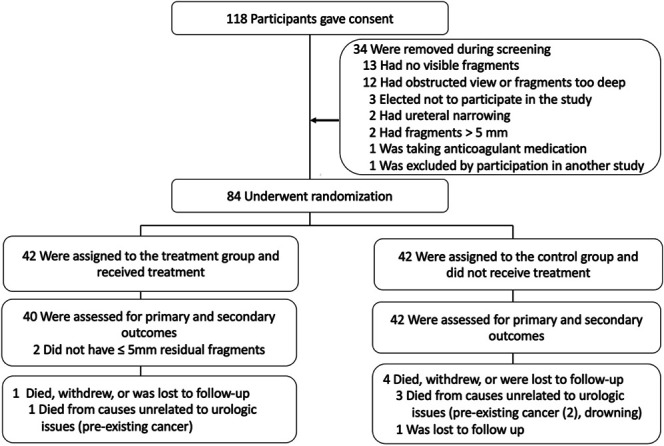
Enrollment and randomization.

The trial was conducted from May 9, 2015, through April 6, 2024, and the last participant was enrolled on May 2, 2023. The study was preempted by 2 other studies recruiting the same patients between 2017 and 2020.^23,24^ The study was paused in 2016 when clinic space was relocated and in 2020 because of the COVID-19 pandemic. A total of 118 patients were enrolled (Figure 1). Thirty-four participants left the study before randomization for the reasons listed. One participant in the treatment group and 3 participants in the control group died from nonurologic causes during the follow-up period. Two participants in the treatment group were excluded because they were found not to have small residual fragments, and 1 participant in the control group was lost to follow-up. All data were included up to the point of death, withdrawal, stone surgery on the study side, or loss of contact with the participant. The group's baseline characteristics (Table 1)^6,25^ were not statistically different (*P* > .1 by Wilcoxon rank sum tests or Fisher’s exact test as appropriate for all characteristics) and representative of the population following up from stone surgery (Supplementary Table S1, https://www.jurology.com).

**Table 1.** Baseline Clinical Characteristics and Follow-Up Data

	Treatment (n = 40)	Control (n = 42)
Age, median (IQR), y	59 (42-72)	62 (51-71)
Female sex, No. (%)	11 (28)	15 (36)
Hispanic ethnicity, No. (%)	4 (10)	5 (12)
Race, No. (%)		
White	37 (92.5)	33 (78.6)
Black/African American	1 (2.5)	2 (4.8)
Asian	0 (0)	4 (9.5)
More than 1 race	1 (2.5)	1 (2.4)
Chose not to report	1 (2.5)	2 (4.8)
BMI, median (IQR), kg/m^2^	27.4 (23.9-31.1)	26.9 (24.7-32.6)
Stone history, No. (%)		
Bilateral stones on preenrollment imaging	22 (55)	20 (48)
Recurrent on enrollment	30 (75)	32 (76)
Taking medical prevention	22 (55)	26 (62)
Primary surgery, No. (%)		
Ureteroscopy	34 (85)	39 (93)
Shock wave lithotripsy	3 (7.5)	3 (7)
Percutaneous nephrolithotomy	3 (7.5)	
Time between surgery and randomization, median (IQR), d	105 (81-140)	114 (80-223)
Target fragment clusters		
Location included lower pole fragments, No. (%)^a^	38 (95)	35 (83)
Left side, No. (%)	31 (78)	25 (60)
Maximum size, median (IQR), mm	6.9 (5.4-9.1)	6.7 (4.1-8.4)
Median clusters, No. (IQR)	1 (1-2)	1 (1-2)
Most recent preprocedure imaging, No. (%)		
CT	21 (53)	25 (60)
Ultrasound	7 (18)	6 (14)
KUB	0 (0)	3 (7)
Ultrasound and KUB at the same time	12 (30)	8 (19)
Fragments identified at some point on a CT exam	29 (69)	32 (76)
Stone composition, No. (%)		
Calcium oxalate	29 (73)	32 (76)
Brushite	2 (5)	1 (2)
Cystine	0 (0)	1 (2)
Struvite	1 (2.5)	1 (2)
Calcium phosphate	2 (5)	1 (2)
Uric acid	1 (2.5)	2 (5)
Other/unknown	5 (13)	4 (10)
Study follow-up, median (IQR), y^b^	3.0 (1.8-3.2)	2.6 (1.5-3.3)

Abbreviations: IQR, interquartile range; KUB, plain film x-ray of kidney, ureter, bladder.

Data were obtained in the medical record.

a Lower pole cases may also have had fragments in other poles as well. Lower pole stones are less likely to pass spontaneously.^6^

b Calculated from nonrelapsing participants following Guidelines for Reporting of Statistics for Clinical Research in Urology.^25^

### Primary Outcome: Relapse

**Figure 2.**
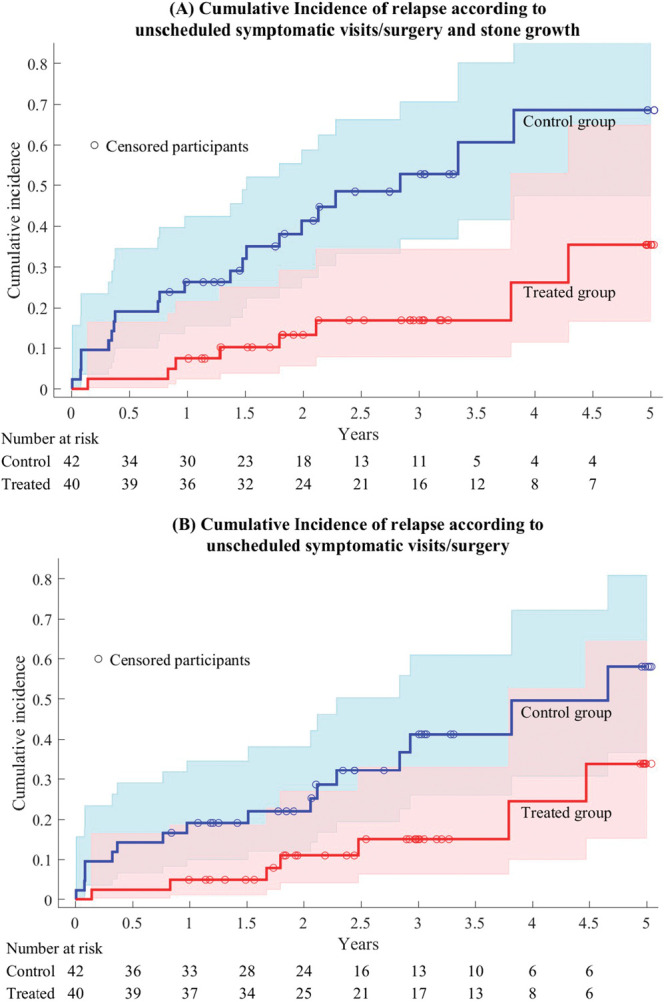
Cumulative incidence of relapse. Shaded areas represent 95% CIs. Small circles represent censored data. Participant relapse data were censored when the participant did not have a relapse before being lost to follow-up or by the end of follow-up.

The primary outcome is shown in Figure 2, A as the cumulative incidence of relapse as measured by stone growth or urgent medical visit or surgery for stones on the study side; by that measure, the control group had shorter time to relapse than the treatment group (*P* < .003 by log-rank test). The restricted mean time to relapse was 1530 ± 92 days for the treatment group and 1009 ± 118 days for the control group. The hazard ratio for relapse in the treatment group, as compared with the control group, was 0.30 (95% CI 0.13-0.68). Relapse events are summarized in Table 2. Relapse occurred in 8 of 40 participants in the treatment group and 21 of 42 participants in the control group.

**Table 2.** Primary Outcome (Relapse) and Secondary Outcome (Fragment Passage)

	Treatment	Control
	n = 40	n = 42
Relapse surgeries, No.	7	10
Relapse unscheduled, symptomatic visits, No.	1	7
Total surgeries or unscheduled, symptomatic visits or both, No.^a^	7	16
Growth of target fragments, No.	4	10
Total surgeries, unscheduled, symptomatic visits, or growth of study stones, No.^b^	8	21
Passed fragments within 3 wk, No. (%)^c^	24 (63)	2 (5)
Passed fragments within 3 wk or saw reduced stone burden on initial follow-up imaging, No. (%)	28 (70)	11 (26)

Four of the 7 (57%) surgeries in the treatment group compared to 2 of the 10 surgeries (20%) in the control group were bilateral. All participants with brushite stones returned for surgery and accounted for 2 of the 7 surgeries (29%) in the treatment group and 1 of the 10 surgeries (10%) in the control group.

a One participant in each group had an emergency department visit and surgery.

b Some participants in both groups had more than one of the following: stone growth; surgery; and urgent, unscheduled, symptomatic visits.

c Two treated participants and 1 control were excluded because they were not asked about passing fragments in the 3 weeks post procedure.

Because of concerns that stone growth could be driving relapse disproportionately, sensitivity analyses were conducted. When stone growth was excluded as a marker of relapse (Figure 2, B), relapse remained greater in the control group (*P* = .03 by log-rank test). The time to relapse was 27% longer in the treatment group than in the control group, with a restricted mean time to relapse of 1574 ± 863 days in the treatment group and 1241 ± 114 days in the control group. An unscheduled, symptomatic clinical visit or surgery—2 of the measures of relapse—occurred in 7 participants in the treatment group and 16 participants in the control group. Eleven participants (28%) underwent more than 1 ultrasonic propulsion procedure for an average of 1.35 treatments per participant. The Supplementary Video (https://www.jurology.com) shows portions of 2 procedures.

### Secondary Outcome: Fragment Passage

Fragment passage within 3 weeks was visually observed in 24 participants (60%) in the treatment group and in 2 participants (5%) in the control group (odds ratio 30.00, 95% CI 6.34-142.00; Table 2). Thirteen treated participants passed stones in clinic at the time of the procedure and 13 shared photographs of fragments vs none of either in the control group.

### Secondary Outcome: AEs

All AEs were mild, transient, and resolved spontaneously except 2 controls reported to the emergency department for renal colic within 90 days (Table 3). Twenty-five participants (63%) in the treatment group and 17 participants (40%) in the control group reported at least 1 AE (odds ratio 2.45, 95% CI 1.01-5.96). Median change in pain scores assessed immediately before and after the procedure for treated participants or screening ultrasound for control participants was 0 of 10. Collectively AEs—hematuria, discomfort, urinary frequency/urgency, dysuria, nausea, and vomiting—were associated with fragment passage by Pearson χ^2^ test (odds ratio 5.81, 95% CI 2.09-16.15) and are not associated with treatment group after adjusting for stone passage in logistic regression.

**Table 3.** Secondary Outcome (Adverse Events)

	Treatment	Control
	n = 40	n = 42
Adverse events, No. (%)		
Serious	0 (0)	0 (0)
Moderate	0 (0)	2 (5)
Mild, potentially related to		
Device or procedure	18 (45)	N/A
Stone disease, not device	7 (18)	17 (40)
Individual mild adverse events, No. (%)^a^		
Skin effects^b^	2 (5)	0 (0)
Hematuria^b^	3 (8)	3 (7)
Discomfort/pain^b^	15 (38)	13 (31)
Urinary frequency/urgency^b,c^	8 (20)	3 (7)
Slow urine flow/incomplete bladder emptying	2 (5)	0 (0)
Constipation	3 (8)	0 (0)
Diarrhea	5 (13)	2 (5)
Dysuria^b^	4 (10)	2 (5)
Nausea^b^	3 (8)	3 (7)
Vomiting	0 (0)	2 (5)
Other^d^	4 (10)	2 (5)

Abbreviations: N/A, not applicable.

a Fourteen participants in the treatment group and 8 participants in the control group had more than 1 adverse event.

b Adverse events potentially related to the device or procedure.

c Six of 8 participants (75%) in the treatment group with urinary frequency/urgency also passed fragments, which can cause urinary frequency/urgency.

d Other adverse events unrelated to the device included COVID-19, malodorous urine, fatigue, and headache in the treatment group, and headache and a feeling of “distended and uncomfortable” in the control group.

Sensitivity analyses including 2 ineligible participants (excluded from an original 42 treatment subjects because they were determined not to have small residual fragments) produced the same log-rank test *P* value for the primary outcome of relapse and similar values for the other measurements; neither reported any AEs. As the intent was to enroll people who had chosen to observe their stones, an additional sensitivity analysis was conducted excluding participants who went to surgery within 90 days of randomization (1 in the treatment group and 2 in the control group); this did not change the results.

## DISCUSSION

In this multicenter, controlled trial, participants with small, residual kidney stone fragments were assigned to the treatment group, in which they received the investigative ultrasonic propulsion procedure to reposition and facilitate passage of their fragments, or to a control group, in which they did not receive the ultrasonic propulsion procedure. We found that repositioning of residual fragments in the treatment group resulted in a 70% lower incidence of relapse, ie, 8 of 40 participants in the treatment group as compared with 21 of 42 participants in the control group. Time to relapse was longer in the treatment group by 521 days (52%). AEs were mild, self-resolving, and similar in both groups. In no cases did a repositioned fragment cause obstruction.

The difference in the number of relapse surgeries (7 vs 10) was not as large as the difference in the number of unscheduled, symptomatic visits (1 vs 7) between the treatment and control groups. However, 4 of the 7 surgeries (57%) in the treatment group and 2 of the 10 surgeries (20%) in the control group were bilateral and thus may have been motivated by the nonstudy side. All participants with brushite stones, known to form new stones quickly, returned for surgery, and accounted for 2 of the 7 surgeries (29%) in the treatment group and 1 of the 10 surgeries (10%) in the control group. These observations suggest potentially an even stronger effect size on relapse rate in favor of the treatment group than captured in our current results.

The study was relatively small, and few of the participants were non-White, which attenuates generalization to other groups. The urologists in this study were aware of the participants’ group assignments during continued care; however, the shared patient care model in their practices, care provided by urologists outside the study, and the high rates of preventive evaluation (over 55% of participants taking medical prevention) in the participating centers likely counterbalanced that limitation. Twelve participants were excluded based on concern that stone depth or interference by ribs or bowel would reduce effectiveness, though in treating some participants, fragments were successfully moved around rib obstruction and at the deepest extent of the imaging (12 cm). Training requirements and user variability were not studied here.^26,27^ However, 9 operators including 5 urologists performed the procedure, and all were able to produce stone movement.

Several studies have shown similar relapse rates in similar control groups,^1-3,28,29^ and several randomized controlled trials have shown noninvasive techniques can reduce stone burden and clear fragments immediately post procedure^8-11^; this paper is a demonstration of long-term clinical benefit of a new technology that was successful at facilitating passage of fragments long after surgery and may have broader applications. When combined with recent evidence that removal of secondary, small, asymptomatic kidney stones during surgery for a primary stone reduced relapse by 82%, these current data suggest ultrasonic propulsion may be an option to remove small stones or residual fragments,^30^ especially when combined with another ultrasound and clinic-based investigational technology, burst wave lithotripsy, to break and expel stones potentially too large to pass asymptomatically.15,^31^ With several studies showing relapse rates of about 50% in 5 years,^1,2^ an office-based procedure such as ultrasonic propulsion may be an attractive, cost-effective way to address asymptomatic stones before they become symptomatic and require urgent evaluation or surgical intervention.

In conclusion, our study showed that removing residual fragments by ultrasonic propulsion reduced relapse as measured by future stone growth or stone-related urgent medical visit or surgery.

## Supplementary Material

SUPPLEMENTARY MATERIAL
