# Supplementary material for: Randomized Controlled Trial of Ultrasonic Propulsion–Facilitated Clearance of Residual Kidney Stone Fragments vs Observation
Source: J Urol. 2024 Aug 15;212(6):811–20. doi: 10.1097/JU.0000000000004186 (PMC11560600; doi:10.1097/JU.0000000000004186)
Supplement: SUPPLEMENTARY MATERIAL [file juro-212-811-s001.pdf]

Supplementary Material

Table of Contents

Table S1. Representativeness of Study Participants	p. 2
References for Table S1	p. 3
Video Caption	p. 4

Table S1. Representativeness of Study Participants

Category	
Disease under investigation	Urinary stone relapse
Special considerations related to	
Sex and gender	Stone disease affects men more than women in the U.S.[1] The odds of kidney stones in females is approximately two-thirds that in men.[1]
Age	Prevalence increases with age.[1] In men and women, the weighted prevalence increases with age; these effects are observed among all racial/ethnic groups.[1]
Race or ethnic group	Stone disease affects white persons disproportionately in the U.S. [1] The prevalence of stone disease is highest among non-Hispanic white individuals, at 10.3%.[1] Among Hispanic individual and among black, non-Hispanic individuals, the prevalence of kidney stones is 6.4 and 4.3%, respectively.[1]
Geography	Stone disease is more prevalent in warmer climates globally.[2,3]
Other considerations	Stone disease is prevalent with 50% recurrence within 5 years.[4,5] Stone disease has some dependence on patient demographics as noted above. The dependence of 1) identification of residual fragments and 2) relapse to an emergency visit or surgery on patient demographics is not known. However, the demographics of those with symptomatic stone disease that are likely to motivate an emergency visit or surgery are known and similar to the demographics of those reporting a history of stones.[1] Nonetheless, different demographic groups may have different access to surgery and medical care, and surgical techniques influence relapse.[6-8] Socioeconomic status is associated with a history of kidney stones.[1]
Overall representativeness of this trial	The participants in the present trial were recruited from subjects following up from surgery and are representative of the demographics of the surgery populations. The study demographics are similar to population rates from the U.S. census weighted by prevalence rate reported in the National Health and Nutrition Examination Survey (NHANES).[1] The study was reasonably representative of demographic groups especially when considering prevalence of stone disease by demographic group. The study tested 2 institutions – a Veterans Affairs health care system and medical center with a university and a county hospital – that each draw from 5 states in the West in an effort to represent U.S. geography.

References for Table S1

1. Scales CD Jr, Smith AC, Hanley JM, *et al*., Urologic Diseases in America Project. Prevalence of kidney stones in the United States. Eur Urol. 2012;62:160-5.
2. Kaufman, J., Vicedo-Cabrera, A.M., Tam, V. *et al.* The impact of heat on kidney stone presentations in South Carolina under two climate change scenarios. Sci Rep 2022;12:369.
3. Romero V, Akpinar H, Assimos DG. Kidney stones: a global picture of prevalence, incidence, and associated risk factors. Rev Urol. 2010;12:e86-96.
4. Assimos D, Krambeck A, Miller NL, *et al*. Surgical management of stones: American Urological Association/Endourological Society Guideline, PART II. J Urol 2016;196:1161-1169.
5. EAU Guidelines. Edn. presented at the EAU Annual Congress Amsterdam 2020. ISBN 978-94-92671-07-3.
6. Ito K, Takahashi T, Kanno T, *et al.*, Decreased recurrence of urolithiasis after simultaneous ureteroscopic surgery for ureter and ipsilateral renal calculi: Comparison to shockwave lithotripsy for ureter calculi alone, Urology 2021;47:74−80.
7. Li, S., Quarrier, S., Serrell, E.C. *et al.*, Should we treat asymptomatic concurrent contralateral renal stones? A longitudinal analysis. Urolithiasis 2022;50:71-77.
8. Djang R, Stahl JE, Pais VM Jr., Informing the management of symptomatic nephrolithiasis: Markov decision analysis for the 1 cm renal stone, Urology Practice 2021;8:495-502.

Supplementary Video Caption

Raw silent video displayed and recorded by the investigational device during portions of two procedures and showing the displacement of residual fragments out of the kidney calyces and in some cases the kidney. The data are cut from larger videos and in one case spliced to other cut segments. The playback speed is the same as the recording speed, and no enhancement has occurred. Additional context has been added around the raw footage with Microsoft PowerPoint software.
